# Ecology of Indigenous Lactic Acid Bacteria along Different Winemaking Processes of Tempranillo Red Wine from La Rioja (Spain)

**DOI:** 10.1100/2012/796327

**Published:** 2012-03-12

**Authors:** Lucía González-Arenzana, Pilar Santamaría, Rosa López, Carmen Tenorio, Isabel López-Alfaro

**Affiliations:** Instituto de Ciencias de la Vid y del Vino (ICVV), CSIC, Universidad de La Rioja, Gobierno de La Rioja, C/Madre de Dios 51, La Rioja, 26006 Logroño, Spain

## Abstract

Ecology of the lactic acid bacteria (LAB) during alcoholic fermentation (AF) and spontaneous malolactic fermentation (MLF) of Tempranillo wines from four wineries of La Rioja has been studied analyzing the influence of the winemaking method, processing conditions, and geographical origin. Five different LAB species were isolated during AF, while, during MLF, only *Oenococcus oeni* was detected. Although the clonal diversity of *O. oeni* strains was moderate, mixed populations were observed, becoming at least one strain with distinct PFGE profile the main responsible for MLF. Neither the winemaking method nor the cellar situation was correlated with the LAB diversity. However, processing conditions influenced the total number of isolates and the percentage of each isolated species and strains. The winemaking method could cause that genotypes found in semicarbonic maceration did not appear in other wineries. Four genotypes of *O. oeni* were isolated in more than one of the rest wineries. These four together with other dominant strains might be included in a future selection process.

## 1. Introduction

Winemaking is defined like the operations and the practises carried out to transform the grapes in wine [1]. This includes two fermentative stages, which are the alcoholic fermentation (AF) led by yeast and the malolactic fermentation (MLF) performed by lactic acid bacteria (LAB). The MLF is not a real fermentation, it mainly consists in a transformation of L-malic acid into L-lactic acid and carbon dioxide, as a part of the LAB metabolism [2], and it contributes to improve the stability and the quality of the final wine [3–5]. The positive effects of MLF in wine depend on the control of the process conditions. *Oenococcus oeni *is the best adapted LAB species to the stressful environment in wine [6, 7] so it is the species which is mostly isolated at this stage [8], being the main responsible for the development of MLF and the most interesting to be selected [9]. In order to get a better control of MLF and to avoid wine spoilage [10, 11], wineries have started to employ commercial cultures from selected *O. oeni* strains. However, not always these malolactic starters are successfully implanted [12]. Several reports have shown that the success of these starters depends on strain and is influenced by several factors, including geographical origin [13] and adaptation to the winemaking conditions of each wine [14–18]. Therefore, it is recommended to study the representative and best-adapted microbiota to the type of wine and the winemaking procedures in each elaboration area. Some authors have conducted studies about ecology of LAB in wineries, but none of them have analyzed more than one winery both at the same vintage in AF and MLF and in the Appellation of Origin Rioja [19, 20]. The main aim of this paper was to analyze the LAB species diversity and the intraspecific diversity of *O. oeni*, studying the geographical distribution at different subzones of this region of the north of Spain. In addition, more relevant correlations between LAB diversity and winemaking process were investigated.

## 2. Materials and Methods

### 2.1. Samples of Musts and Wines

**Figure 1:**
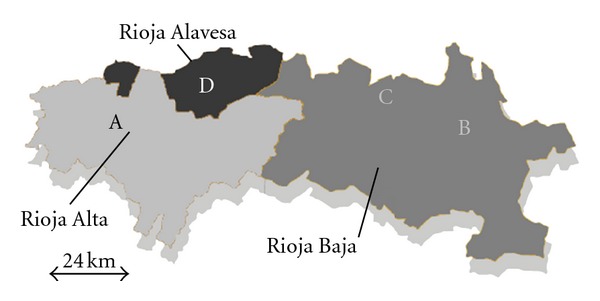
Location of the four wineries in the three subzones of the Appellation of Origin Rioja.

Samples of Tempranillo red wine were taken from four wineries located in three different subzones of Appellation of Origin Rioja (Figure 1). None of the surveyed wineries had ever used LAB commercial starter cultures. The AF was carried out by the destemming and crushing method in stainless-steel tanks, except in the case of winery D which was carried out by the traditional semicarbonic maceration method (whole grape) in open cement tanks.

When AF was completed, wines were racked and placed in stainless-steel tanks in the case of wineries A, B, and C and in open cement tank for winery D. The wines underwent spontaneous MLF with the endogenous microbiota (no starter inocula was used).

One fermentation tank was sampled in each winery. Wine samples were taken aseptically for chemical and microbiological analysis at different times: must (stage 1), tumultuous AF (density around 1,025; stage 2), at the end of AF (<2 g/L glucose + fructose; stage 3), initial MLF (consumption of 10% of the initial malic acid; stage 4), tumultuous MLF (consumption of 60% of the initial malic acid; stage 5), and at the end of MLF (L-malic acid concentration <0.5 g/L; stage 6). Wineries A and B were only sampled after the end of AF.

### 2.2. Chemical Analysis

Alcohol degree, pH, total acidity, volatile acidity, reducing sugars, free and total sulphur dioxide (SO_2_), tonality, and colour intensity were measured according to the European Community Official Methods [21]. Histamine was analyzed by reverse-phase HPLC using the method reported by López et al. [22]. MLF was followed by measuring wine L-malic and L-lactic acid content by enzymatic methods [21] (Enzymatic BioAnalysis, Boehringer-Mannheim/R-Biopharm, Germany).

### 2.3. Bacterial Enumeration and Isolation

Samples were diluted in sterile saline solution and plated on MRS agar (Scharlau Chemie S.A., Barcelona, Spain) plates supplemented with tomato juice (10% v/v), fructose (6 g/L), cysteine-HCl (0.5 g/L), L-malic acid (5 g/L), and 50 mg/L of pymaricine (Acofarma, S. Coop., Spain). Plates were incubated at 30°C under strict anaerobic conditions (Gas Pak System, Oxoid Ltd., Basingstoke, England) for at least ten days, and viable counts were reported as the number of CFU/mL. Fifteen colonies from each wine sample were selected for reisolation and identification. Isolates were stored in 20% sterile skim milk (Difco) at −20°C.

### 2.4. Species Identification

Species identification was carried out by previously recommended methods, which included bacteria morphology, Gram staining, and catalase reaction [23]. In order to get a better and more precise identification, molecular biology methods were used. *Oenococcus oeni*, *Lactobacillus plantarum*, and *Lactobacillus brevis* species were confirmed by the species-specific PCR method [24, 25]. In case of identification of other unknown species, PCR amplification of partial 16S rRNA genes was performed with WLAB1 and WLAB2 as previously described López et al. [26]. PCR products were sequenced by Macrogen Inc. (Seoul, Republic of Korea), and sequences were used for comparison to the data in GenBank using the Basic Local Alignment Search Tool (BLAST) [27].

### 2.5. Strain Typing of *O. oeni*

PFGE was carried out according to the method described by Birren and Lai [28], with some modifications [8] for agarose block preparation. Macrorestriction analysis was performed with two endonucleases, first with *Sfi*I following the method reported by López et al. [8] and then with *Apa*I by the method reported by Larisika et al. [29], with modifications for optimal separation of fragments: 1.2% (w/v) agarose gels were submitted to 24 h with a pulse ramping between 0.5 and 20 s at 14°C and 6 V/cm in a CHEF DRII apparatus (Bio-Rad).

### 2.6. Numerical Analysis of Gel Images

The conversion, normalization, and further processing of images were carried out by FPQuest software version 5.1 (Bio-Rad, USA). Comparison of the obtained PFGE patterns were made by Pearson correlation coefficient and with Unweighted Pair Group Method using Arithmetic Averages (UPGMA).

## 3. Results and Discussion

Results for analytical composition of wines in the four studied cellars are displayed in Table 1. Data were within the range of Tempranillo wines from this Spanish region [22]. In all the wineries, volatile acidity underwent a light increase after MLF as expected, and, in any case, it was important to the final quality of the wine. The wine colour intensity and total phenols decreased after MLF, and the tonality increased slightly in all the wineries. Histamine in wines increased during MLF, but its concentration was low in all the studied wines, except in winery A. Different factors usually affect the biogenic amine formation [30]; the main is the presence of free amino acid and microorganisms able to decarboxylate them. This ability is highly variable, and it depends not only on the species but also on the strain and on the environmental conditions [31].

**Figure 2:**
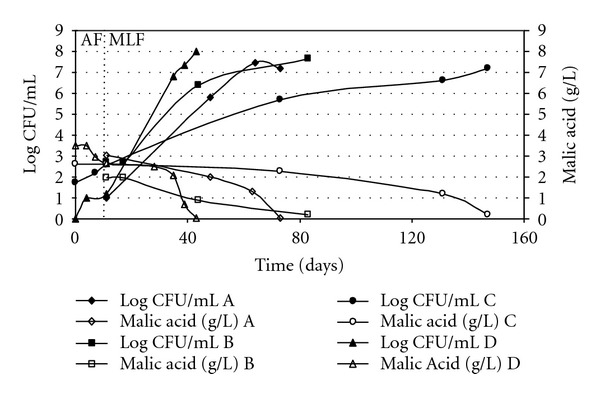
Viable LAB population and L-malic acid concentration in winemaking.

Figure 2 shows the evolution of the viable LAB population and the L-malic acid decrease during the fermentation period. In every situation, an increase in LAB population was related to decrease levels of L-malic acid. The AF in wineries C and D was developed in 11 days. The MLF had variable duration ranged between 32 days in cellar D and 136 days in cellar C; this longer fermentation was probably due to the lower pH and lower temperatures of the wine at the end of AF in this winery. In spite of the low temperatures, this winery did not use a control temperature method during MLF, what could produce longer latent period [9, 14, 32, 33]. The LAB viable populations were in the range of 1.5 × 10^1^–1.4 × 10^3^ CFU/mL at the end of AF and 1.4 × 10^6^–3 × 10^7^ CFU/mL at the end of MLF, similar to other spontaneous MLF [20, 34].

Table 2 shows the percentage of LAB species identified at each fermentative stage in every sampled winery. The highest species richness was detected in winery B in which five different LAB species were identified. *O. oeni* was not isolated at stage 4 in this winery, what could be caused as the MLF had not actually begun as it was indicated by the viable LAB count at this stage (Figure 2). Three and four LAB species were, respectively, found in cellars C and D, while in A only *O. oeni* was isolated.

Data of species distribution along winemaking showed that highest number of LAB species was observed during AF. In wineries A and C, *O. oeni* became the main LAB isolated by the end of AF and it was the only one during MLF; these results have already been reported by other authors [13, 15, 29, 33] which highlighted the enormous adaptation of *O. oeni* to the strict wine conditions [16, 20].

Apparently, neither the type of winemaking nor the cellar situation was correlated with the diversity of LAB species in each cellar. However, processing conditions in each winery could influence the total number of isolates and the percentage of each isolated species. Thus, higher SO_2_ levels in AF could favour the growth of more resistant species to this antiseptic [35] (wineries B and D) and lower pH could promote the *O. oeni *development [32] (winery C).

Information relating to *O. oeni* typing is covered in Table 3. After subjecting the 182 *O. oeni* isolates to PFGE with *Sfi*I endonuclease, twenty-eight different genotypes were detected (data not shown). Analysis with a second enzyme (*Apa*I) did not increase the number of differentiated patterns. Wineries B and D showed five distinct patterns for each one, A showed eight, and C fifteen. To our knowledge few studies of *O. oeni* strain variability during both AF and MLF have been reported so far. After comparing strain diversity between wineries, similar and moderate indexes of diversity (ID) [36] were observed in wineries A, B, and D in MLF. No correlation between geographical situation and strain diversity was observed as has been reported to yeast by Santamaría Aquilúe [37]. However, the vinification conditions could have greatly influenced strain diversity. Thus, winery C showed a higher ID in AF which decreased considerably in MLF. This decrease could be due to the lower fermentative temperatures which caused that a lower number of genotypes were adapted to those conditions. Moreover, in winery D, the use of higher sulphite concentrations could determine the low LAB populations found during AF, so quantifying the ID of *O. oeni* was not possible at this fermentative period.

Table 4 includes the information about the twenty-eight *O. oeni* genotypes and their frequency (%) of appearance in each winery. Most of the genotypes (eighteen) appeared only at one stage during the vinification with frequencies from 1% to 15%, while the rest of genotypes (ten) were isolated at more than one stage, representing highly variable frequencies of appearance from 3% to 55%. Only three patterns (genotypes 10, 11, and 24) were isolated at both AF and MLF.

The frequency of participation of each genotype varied from winery to winery, so dominant ones in one cellar were minority or not present at other one. Thus, genotype 3 was detected in wineries A and C; genotypes 11 and 12 were isolated in wineries B and C, while the pattern 10 was identified in three wineries (A, B, and C) that were further away (Figure 1). However, their frequency of appearance was extremely variable at each winery. Thus, for example, genotype 12 reached the 45% of the total *O. oeni *isolates in cellar C, it was the 9% in B and not being isolated in the other two wineries. This fact proved that although some genotypes appeared in more than one winery, they were not always the majority *O. oeni* strain in spontaneous MLF, because their frequency depended on the elaboration conditions and on the wine composition. Thus, different strains were the best adapted and performed MLF in each winery. On the other hand, the total five genotypes detected in winery D were not present in the others. It could be related to the different type of winemaking used in this winery (open cement tanks with whole grapes) what might create a special ecosystem with an own microbiota.

Curiously, the two majority genotypes (patterns 10 and 12) found in the wine from cellar C were indistinguishable to the ones detected in the sampled air of the same winery in a study made by Garijo et al. [38, 39] at a previous vintage.

**Figure 3:**
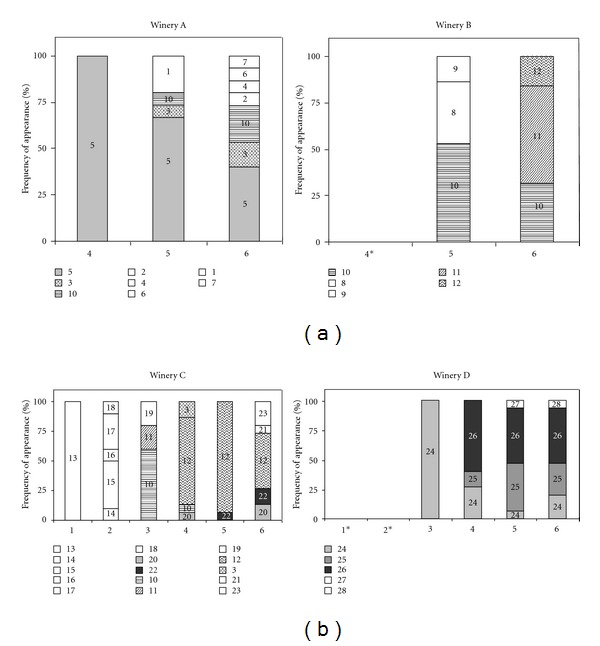
Frequency of appearance (%) of *O. oeni* genotypes at each sampled stage (1: must; 2: tumultuous AF; 3: final AF; 4: initial MLF; 5: tumultuous MLF; 6: final MLF) in each winery. Empty bars mean genotypes that appeared once, filled bars mean genotypes that appeared in one winery, and textured bars were genotypes detected in more than one winery. *O. oeni was not detected.

Figure 3 shows the distribution of the detected *O. oeni *genotypes at the analyzed stages in the four wineries. At most stages, in both AF and MLF, mixed *O. oeni* populations were observed, so there were different genotypes able to share their ecological niche or tank, as other authors have described in MLF [8, 20]. The number of different identified genotypes at each stage ranged from 0 to 5 and from 2 to 7 during AF and MLF, respectively. In this study, it was also observed that, between all the genotypes present in the same tank, there was at least one detected during all the MLF process and in a bigger percentage, thus it would be the responsible for the MLF. In some cases, this main genotype only appeared in one cellar, such as pattern 5 and 26, but, in others, it was present in more than one winery as patterns 10 and 12.

In summary, this study is a contribution to a better description of the LAB ecology along the process of Tempranillo wines winemaking. The conditions of elaboration along with the winemaking method influenced the microbial diversity of LAB populations either at species and strain level. *O. oeni* became the main identified species, and a complex diversity of indigenous *O. oeni* strains was observed with genotypes that were relaying each other along the process. This diversity was moderate in MLF so one or two patterns became majority. Genotypes at each winery sometimes were distinctive at each one, and others were coincident between wineries. The four genotypes that were isolated in more than one winery in highly variable frequency could be the result of a successful adaptation to each particular winemaking condition. Convergence in winemaking ecology, due perhaps to the adaptation of strains to the stressful conditions in wine fermentation or perhaps to dispersion factors, such the air, birds, or insects [40], might explain the presence of these four indistinguishable genotypes in wineries that were far apart and in the air of one of these cellars. Dominant genotypes (except pattern 5) can be considered as interesting *O. oeni* strains to be included in a selection process, and they would contribute to preserve the biodiversity and peculiarity of the wine. The aminobiogenic capacity of LAB strains used as starter cultures for MLF should be tested prior to strain selection; thus pattern 5 would result a problematic strain because it persisted as majority in MLF of a wine with a high histamine level. Further investigation must be expanded to more wineries and vintages, including vineyards in order to clarify the aspects that have shaped ecology of the wine LAB in this region.

## Figures and Tables

**Table 1:** Analytical composition of wines at final AF (stage 3) and final MLF (stage 6) in each winery.

Winery	A		B		C		D	
Stage	3	6	3	6	3	6	3	6
Alcohol content (% v/v)	12.9	—	13.4	—	14.0	—	12.4	—
pH	3.56	3.73	3.50	3.59	3.32	3.50	3.61	3.86
Total acidity (g/L tartaric acid)	7.69	5.81	7.72	6.63	9.00	7.20	5.62	5.49
Volatile acidity (g/L acetic acid)	0.22	0.40	0.33	0.45	0.26	0.37	0.16	0.30
L-malic acid (g/L)	3.02	0.05	1.97	0.19	2.61	0.21	2.44	0.05
L-lactic acid (g/L)	—	1.40	—	1.21	—	1.72	—	1.76
Free SO_2_ (mg/L)	14.5	—	17.0	—	13.2	—	29.8	—
Total SO_2_ (mg/L)	31.6	—	44.4	—	31.6	—	47.6	—
Total phenols (OD 280 nm)	53.2	48.8	66.1	53.1	71.3	67.0	63.5	57.9
Colour intensity (OD [420 + 520 + 620] nm)	13.5	8.30	20.1	10.1	29.1	27.8	15.0	10.2
Tonality (OD 420/520 nm)	0.44	0.60	0.41	0.57	0.34	1.26	0.40	0.54
Histamine (mg/L)	0.00	6.52	0.00	3.39	0.00	0.33	0.00	0.33

(—) not analyzed.

**Table 2:** Percentage of the LAB species at each stage of the vinification in the four studied wineries.

Winery	A			B			C						D					
Stage*	4	5	6	4	5	6	1	2	3	4	5	6	1	2	3	4	5	6
*O. oeni*	100	100	100	—	100	100	100	77	100	100	100	100	—	—	33	100	100	100
*L. plantarum*	—	—	—	36	—	—	—	8	—	—	—	—	—	50	—	—	—	—
*L. mali*	—	—	—	50	—	—	—	15	—	—	—	—	—	—	—	—	—	—
*Ln. mesenteroides*	—	—	—	7	—	—	—	—	—	—	—	—	—	50	—	—	—	—
*Lactobacillus sp.*	—	—	—	7	—	—	—	—	—	—	—	—	—	—	67	—	—	—

*1: must; 2: tumultuous AF; 3: final AF; 4: initial MLF; 5: tumultuous MLF; 6: final MLF.

(—) not detected.

**Table 3:** Number of isolates and genotypes of *O. oeni* and index of diversity (ID*) during AF and MLF.

Winery	A	B	C		D	
Stages	MLF	MLF	AF	MLF	AF	MLF
N° of total isolates	31	44	29	45	5	45
N° of *O. oeni* isolates	31	30	26	45	1	45
N° of *O. oeni* genotypes	8	5	9	7	1	5
ID*	0.65	0.66	0.84	0.49	—	0.65

*ID = 1 − [1/*N*(*N* − 1)]∑*n*_j_(*n*_j_ − 1), where the number of strains is *N*, and *n* is the strains belonging to type “j.”

(—) incalculable.

**Table 4:** *O. oeni* genotypes, isolation stage, and frequency^a^ (%) of their appearance in each winery.

Genotypes	Isolation stage^b^	Wineries			
		A	B	C	D
1	5	10			
2	6	3			
3	4-5-6	10		3	
4	6	3			
5	4-5-6	55			
6	6	3			
7	6	3			
8	5		15		
9	5		6		
10	3-4-5-6	13	41	14	
11	3-6		29	4	
12	4-5-6		9	45	
13	1			1	
14	2			1	
15	2			6	
16	2			1	
17	2			4	
18	3			1	
19	3			4	
20	4-6			4	
21	6			1	
22	5-6			4	
23	6			4	
24	3-4-5-6				20
25	4-5-6				26
26	4-5-6				50
27	5				2
28	6				2

^a^% Appearance = *n*° strains with a specific PFGE pattern × 100/total *n*° of isolates per winery.

^b^1: must; 2: tumultuous AF; 3: final AF; 4: initial MLF; 5: tumultuous MLF; 6: final MLF.
